# The Way Calories Are Displayed on Restaurant Menus May Not Affect Calorie Intake: Evidence from an Online Experiment

**DOI:** 10.3390/nu17233642

**Published:** 2025-11-21

**Authors:** Riccardo Migliavada, Michele Ricci, Giulia Garavelli, Federica Zoe Ricci, Luisa Torri

**Affiliations:** 1 University of Gastronomic Sciences of Pollenzo, Piazza Vittorio Emanuele II, 9, 12042 Pollenzo, Italy; 2 Department of Statistics, University of California Irvine, Irvine, CA 92697-1250, USA

**Keywords:** calorie labeling, consumer behavior, nutrition knowledge, Physical Activity Calorie Equivalent (PACE), Percent Daily Intake (PDI)

## Abstract

**Background/Objectives:** Menu calorie labeling policies aim to promote healthier eating habits, yet their effectiveness remains debated. The present study aimed to evaluate the effectiveness of two alternative qualitative labeling strategies—Physical Activity Calorie Equivalent (PACE) labels and Percent Daily Intake (PDI) pie charts—compared to the standard numeric calorie count mandated in several countries, since they have been proposed to enhance consumer comprehension and decision-making. **Methods:** A nationwide online survey elicited responses from N = 885 individuals living in Italy. Survey participants were randomly assigned to one of three menu conditions: (1) numeric calorie labeling only, (2) numeric calories plus PACE labels, or (3) numeric calories plus a PDI pie chart. Participants selected a three-course meal from their respective menus. Data on sociodemographic factors, dietary habits, BMI, self-assessed nutritional knowledge, and psychological traits—restrained eating (DEBQ-R) and impulsivity (SUPPS-P)—were collected. Ordinal logistic regressions assessed the impact of labeling format, gender, and nutritional knowledge on total calories ordered. **Results:** Neither PACE labels nor PDI pie charts significantly influenced total calorie selection compared to numeric calorie labels alone. No significant interactions emerged between labeling format and gender or nutritional knowledge. However, age, BMI, and dietary habits were strongly associated with calorie choices: older adults, individuals with restrictive diets, and those with higher restrained eating scores selected lower-calorie meals, while participants with higher BMI or frequent meat intake opted for more calorie-dense options. **Conclusions:** Alternative calorie labeling formats alone may be insufficient to alter food choices in online settings. Future interventions should integrate motivational and educational strategies tailored to individual traits and dietary habits, rather than relying solely on calorie presentation format.

## 1. Introduction

In recent decades, obesity and overeating have become major social burdens in many countries, impacting individual health and well-being while representing substantial costs to healthcare systems and state finance [1,2]. As a response, many governments have implemented policies aimed at promoting healthier eating behaviors [3,4]. Among these policies, a popular, yet debated intervention is to require calorie counts, expressed in kilocalories (kcal) or kilojoules, to be displayed next to items’ names or prices, on restaurant physical menus, online menus, and menu boards. Calorie labeling is often accompanied by a statement regarding the recommended daily calorie intake for an adult (e.g., 2000 calories a day is used for general nutrition advice, but calorie needs vary).

The United States, Australia, and Canada are among the countries that require restaurants and cafeterias with 20 or more locations to display the calorie content of all food and beverage items on their menus. In the United Kingdom, mandated menu calorie labeling applies to businesses with 250 or more employees.

The rationale behind mandatory calorie information on restaurant menus—often referred to as menu calorie labeling- is that consuming an appropriate amount of calories is essential for maintaining a healthy body weight. However, while restaurant foods tend to be higher in calories compared to homemade meals [5], consumers often struggle to estimate the calorie content of restaurant foods accurately, with “caloric illiteracy” (i.e., the lack of awareness, understanding, or ability to accurately estimate the caloric content of foods) identified as a significant factor in the global increase in obesity rates [6]. It appears, therefore, important that, when eating out, consumers are provided with clear and accessible calorie information. Hence, menu calorie labeling has been proposed as a cost-effective intervention to promote long-term public health [7].

Although this strategy could intuitively appear effective, evidence of its actual impact is mixed. Some studies have found a moderate reduction in calories ordered due to calorie labeling [8,9,10,11,12], while others observed no significant change [13,14,15,16] or reported contradictory outcomes [17,18].

One key reason for mixed results seems to be the visibility of calorie labels. Studies report effectiveness primarily among consumers who actively notice the calorie information [19,20]. A recent survey by [21] revealed that only 52.7% of respondents noticed calorie labels during their last meal out [22]. However, making calorie labeling more prominent (e.g., using larger, bold text) does not seem to be enough [23]. Findings also suggest that including contextual or interpretive nutrition information along with calories might help consumers to select lower-calorie meals when eating out [24]. Thus, improving the clarity of calorie information is critical.

A growing body of research has investigated qualitative labeling systems as alternatives or complements to numeric calorie counts to increase readability and ease of use.

These approaches include the use of interpretive symbols, graphs, and intuitive representations that do not require numeric calculations, making nutritional information more immediately understandable [25,26,27]. Studies also show that symbolic calorie labels can appeal to a broader demographic, reducing calorie intake of consumers with varying levels of health awareness [28].

Within qualitative labeling, the Physical Activity Calorie Equivalent (PACE) label, which indicates the exercise (in minutes or miles/km) needed to burn off a dish’s calories, stands out as particularly effective and user-friendly [29]. Compared to other labeling methods, PACE labels are more likely to capture attention and have been linked to a reduction in high-calorie food purchases [30,31]. PACE labeling may also encourage health-conscious decision-making, nudging consumers toward lower-calorie items [32]. From a behavioral economics perspective, PACE labels are expected to influence decisions because they translate an abstract, numerically framed calorie value into a concrete “effort cost” expressed as minutes of physical activity. By linking food choices to the amount of effort required to “burn off” the calories, PACE labels may heighten the perceived personal relevance of energy content and evoke aversion to unnecessary effort. This label format appears particularly effective for future-oriented individuals who demonstrate more self-control over caloric intake [33]. However, not all studies confirm its effectiveness, especially in real-world contexts, where results appear less consistent than those obtained in controlled settings [34,35]. PACE labeling has also been criticized both for failing to distinguish between high and low energy density calories and for the potential risk of exacerbating eating disorders (for a list of issues related to PACE labels, see [36]).

Research has shown that combining calorie information with Percent Daily Intake (PDI) can significantly reduce caloric intake in specific contexts, such as fast-food menus, compared to calorie information alone [37]. By visually depicting the proportion of recommended daily energy intake represented by a single meal, these labels may facilitate proportional reasoning and intuitive impressions (e.g., whether a meal takes up a “small”, “moderate”, or “large” share of daily energy needs) without requiring complex calculations. In this sense, PDI labels could lower the cognitive load associated with interpreting calorie information and support more accurate and immediate impressions of whether a given option is “too much” relative to daily energy recommendations. This suggests that contextual and interpretive formats like PDI can enhance numeric calorie counts by placing them within broader dietary goals. Additionally, studies on front-of-pack (FOP) label design emphasize the importance of visual placement and format in maximizing effectiveness, with PDI formats emerging as particularly effective in capturing consumer attention and influencing food choices [38]. However, other studies have found no significant difference in the calories ordered between menus with no calorie information and those using PDI [39].

This mixed evidence highlights the need for more accessible and intuitive methods to present PDI information, such as pie charts. Pie charts combine visual prominence and intuitive understanding, making them a promising complement to calorie information on menus. They are quick and easy to interpret, requiring minimal cognitive effort from consumers [40], and have been shown to improve the accuracy of estimating limits for weekly servings [41]. Thus, integrating PDI into a pie chart format may leverage its strengths, offering consumers a tool that is both engaging and effective in promoting healthier food choices.

A survey of US public preferences revealed that consumers expressed nearly equal support for numeric calorie counts, PACE labeling, and percentage of total energy intake information [42]. However, the comparative effectiveness of these two approaches remains underexplored. While PACE appeals to the consumer’s intuitive sense of effort and energy expenditure, PDI pie charts focus on providing a clear, visual snapshot of how a food item fits into overall dietary goals. This complementary nature suggests that both labeling systems seek to bridge the gap between raw numeric data and actionable insights, making calorie information more accessible and meaningful. The direct comparison of these two systems, alongside standard calorie labeling, would provide critical insights into which qualitative approaches are most effective in helping consumers make healthier food choices.

Individual characteristics, such as gender, age, education, socioeconomic status, body mass index (BMI), and personality traits, can influence how individuals respond to caloric information, as demonstrated by some studies [21,28,42,43]. Regarding gender differences, previous research has demonstrated that women exhibit greater sensitivity to nutritional information compared to men [44], are more likely to notice calorie information [21], and order fewer calories than men when exposed to calorie labels [31].

Nevertheless, in some studies, male participants appeared more likely to answer correctly to questions on the meaning of calories, which may influence their dietary choices [31]. A survey of the American population conducted by [42] revealed that women were significantly more likely to report that calorie posting would encourage them to select lower-calorie options. Furthermore, they were significantly more likely to report feeling guilty about choosing higher-calorie foods when calorie information was displayed on the menu. Women also exhibited a significantly higher likelihood than men of paying attention to calorie information when it was presented alongside price on the menu.

These findings may reflect broader gendered patterns in food-related attitudes and behaviors. As suggested by [45], women tend to be more engaged with food-related issues due to stronger associations with health, body image, and ethical concerns, which may amplify their attention and responsiveness to nutritional cues. Thus, gendered engagement with food is likely a contributing factor—not merely an individual preference—shaped by sociocultural norms and expectations. However, few studies have explored whether this sensitivity might amplify or reduce the impact of qualitative approaches. Understanding these dynamics is essential to identifying approaches that are effective across genders.

Another key factor in the effectiveness of labeling is consumers’ nutritional knowledge. Individuals with higher levels of nutritional knowledge are generally more likely to use nutritional information to make healthier choices [19]. Conversely, systems like PACE might be more accessible to individuals with lower nutritional knowledge, thanks to their simplicity and immediacy. On the other hand, pie charts could be more effective for those who are better equipped to interpret visual data and contextualize nutritional information relative to their daily needs. However, contradictory results have been reported in the literature. Ref. [28] reported that calorie labels have the greatest impact on individuals who are least health conscious, while according to [17], calorie information may decrease the calories ordered by health-oriented individuals with higher nutritional knowledge. Similarly, Ref. [46] reported that PACE label appeared to be more influential in motivating food choices among individuals with adequate health literacy. While the existing literature provides some insights into the role of consumer knowledge in label effectiveness, the current evidence base is inconclusive. Thus, given the potential for labeling to influence dietary choices, a deeper understanding of how consumer knowledge interacts with label design is crucial for developing effective public-health interventions.

Socioeconomic status also plays a role in determining the effectiveness of calorie labeling, with individuals possessing higher levels of health literacy benefiting more from such information [47]. However, other research has found no significant effect of these sociodemographic characteristics on individuals’ responses to caloric information [34,35]. This inconsistency suggests that the impact of individual characteristics on how people react to caloric information may be complex and that it can vary depending on other factors.

Concerning personality traits, impulsivity and restrained eating habits influence food choices in a restaurant context [48]. For instance, impulsive individuals are not only more prone to overeating [49] but they are also more likely to gravitate toward high-calorie foods [50] and show an attentional bias toward these options [51]. In contrast, restrained eaters tend to focus more on menu calorie information and are likelier to choose lower-calorie options compared to their unrestrained counterparts [52,53].

Although qualitative menu labeling is gaining support among scholars and policymakers, the optimal way to present calorie information remains debated and empirically unresolved [54]. Furthermore, calorie labeling in general—despite its widespread adoption as a policy instrument—has been criticized for its limited real-world impact and for failing to account for the multifaceted determinants of food choice [55,56]. Indeed, eating decisions are rarely based solely on nutritional data; they are influenced by a complex constellation of factors, including taste preferences, price, habits, cultural norms, emotional states, and social contexts [57,58]. Labels, especially numeric ones, often occupy a marginal role in the decision-making process.

Critically, some scholars argue that labeling policies may inadvertently exacerbate inequalities and have a negative impact on people with eating disorders [47,59]. While our study does not attempt to capture the full range of motivations behind food selection, we recognize these limitations and share the view that calorie labeling—particularly in its current, numeric-only form—offers at best a partial and insufficient response to diet-related public health challenges.

Nonetheless, given that many governments have already implemented mandatory calorie labeling policies—and others are actively considering them—we believe it is essential to assess whether and how such interventions can be improved. Rather than endorsing calorie labeling as an effective or comprehensive tool, our aim is to critically evaluate whether enhanced, more intuitive formats (e.g., PACE and PDI pie charts) offer greater usability and behavioral impact, especially when accounting for individual differences such as gender, nutritional knowledge, impulsivity, and restrained eating. Understanding these interactions may inform refinements to existing policies and support more targeted, equitable approaches.

In this context, the present study aimed to evaluate the effectiveness of two qualitative labeling approaches—PACE labels and PDI pie chart—compared to the standard numeric calorie count mandated in several countries. Through a large-scale survey of the Italian population, we examined (1) the impact of calorie labeling on the overall caloric intake, (2) how respondents’ gender influenced the effectiveness of different labeling formats, (3) whether nutritional knowledge shaped calorie choices across these formats, and (4) how impulsivity and restrained eating moderated labeling impact. By incorporating sociodemographic and psychological factors, this research sought to provide deeper insights into the contexts and conditions under which calorie labeling can effectively support informed decision-making and encourage healthier eating behaviors. At the same time, the study explicitly recognizes the limitations of calorie labeling as a standalone intervention and positions its findings within a broader critical discourse on the real-world relevance, usability, and risks of such public health tools.

## 2. Materials and Methods

Our study is based on an online survey, whose structure is described in detail below, in Section. The survey was developed in Italian and distributed via email and social media (i.e., LinkedIn, WhatsApp, Facebook) over a period of one month. The target of the survey were individuals living in Italy at the time of data collection, allowing us to investigate food choices in a context not yet influenced by mandatory calorie labeling on restaurant menus. Participants were informed that the aim of the study was to investigate consumers’ perception of restaurant menus. The survey was designed and administered using the online platform Qualtrics (Provo, UT, USA).

The survey received a total of 1047 responses. Of all responses collected, 162 were excluded due to the following reasons: nine due to a decline of consent; 96 responses were left blank, and ten missed required information; 47 responses were discarded because the respondents did not live in Italy. The final sample consisted of responses from 885 study participants, all of whom provided their written informed consent at the beginning of the survey.

The study was approved by the Ethics Committee of the University of Gastronomic Sciences (Ethics Committee Proceeding n. 2024.02) and conducted according to the guidelines of the Declaration of Helsinki.

### 2.1. Data Collection

Data were collected anonymously through an online self-reported survey composed of 46 questions and approximately 10 min long. The following five sections were included in the survey: (i) meal selection from a restaurant menu; (ii) demographic characteristics, height, weight, and nutrition; (iii) restrained eating assessment; (iv) impulsivity assessment; (v) income (non-mandatory). An English translation of the original survey can be found in Supplementary Materials S1.

#### 2.1.1. Meal Selection from a Restaurant Menu

At the beginning of the first section of the survey, participants were asked to state their Hunger level on a 7-point scale (1 = Not at all hungry; 7 = Extremely hungry) developed by [60]. Next, participants were asked to imagine being in a fictitious restaurant and choosing the dishes they were most willing to order from the restaurant’s menu. Survey respondents must choose a meal composed of three courses: an entrée, a side dish, and a dessert. They could choose among four entrée options (Pizza Margherita, Lasagna, Red rice salad, Vegan burger), four sides (Mini corn cobs, Chicken nuggets, Cheesy triangles, Green salad), and four desserts (Tiramisu, Brownie, Fruit, Soy yogurt). Each course—main and sides—included one animal-based option, while the remaining three items were either vegetarian or vegan. Desserts included two vegan and two vegetarian options, ensuring a broad representation of plant-based choices across all menu categories. The food items included in the menu were selected in order to reflect products currently offered by the most prominent fast-food chains operating in Italy, and the calorie values were estimated based on similar items listed on their menus.

**Figure 1:**
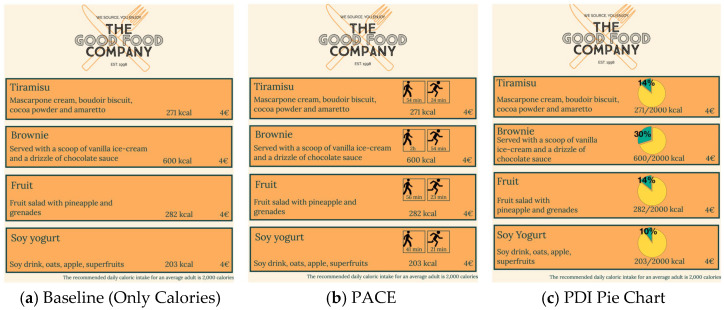
Appearance of three menu designs that participants were randomly assigned to visualize. Like the Baseline (Only Calories) menu (**a**), all menus indicated the number of calories for each dish (next to the dish description) and the number of daily recommended calories (at the bottom right of the page). The PACE menu (**b**) also displayed the minutes of walking and running required to burn the amount of calories in the dish (above the number of calories). The PDI Pie Chart menu (**c**) displayed the number of calories as a proportion of the recommended daily caloric intake for an average adult, and illustrated the proportion through a pie chart.

For each course, as shown in Figure 1 for the desserts course, the menu stated the name of available dishes, the dish description, and the price. To minimize the effect of price on choices, all entrées were priced at 10 euros, all sides were priced at 6 euros, and all desserts were priced at 4 euros. Participants were randomly assigned to visualize one of the three different menu designs. The three menu designs were identical in terms of dishes offered, portion size, and price information, but differed in terms of the way calories in the dishes were displayed. All menus presented the number of calories for each dish (Figure 1a); the second menu included also PACE labels stating the minutes of walking and running required to burn the amount of calories in the dish based on the work of [61] (Figure 1b); the third menu stated the number of calories as a proportion of the recommended daily caloric intake for an average adult, and illustrated the proportion with a pie chart (Figure 1c). All menu designs presented the statement “The recommended daily calorie intake for an average adult is 2000 calories” at the bottom, as mandatory in several countries. The calorie values provided for the foods included in the menus were estimated based on the values of similar items listed on the menus of major fast-food chains operating in Italy.

#### 2.1.2. Demographic Characteristics, Anthropometric Measurements, and Nutrition

The second section of the survey asked participants the following questions about their demographic characteristics and anthropometric data: age (in years), gender (Male, Female, Other, Prefer not to declare), height (in centimeters), body weight (in kilograms), level of education attained (Lower than high-school diploma, High-school diploma, Bachelor’s degree, Master’s degree, Doctorate), country of nationality, country of residence and location of residence (City, Suburbs, Countryside).

Participants were then asked questions related to their nutrition: their self-assessed nutritional knowledge (i.e., How well informed do you consider yourself in the nutritional field?, rated on a 5-point Likert scale with 1 = Not at all and 5 = Completely informed); their frequency of dining out (Never, Rarely, Sometimes, 1–3 times per month, 1–2 times a week, 3–4 times a week, 5 or more times a week); their diet (Vegan, Vegetarian, Flexitarian, Pescetarian, Omnivorous, Other); whether they were following a weight-loss diet.

#### 2.1.3. Restrained Eating Questionnaire

The third section of the survey was a version of the self-reported Dutch Eating Behavior Questionnaire (DEBQ), developed by [62] and translated to Italian by [63]. The restrained scale of the DEBQ (DEBQ-R) used in this work is composed of 10 items, of which three assess the sub-scale of Restrained intention and seven assess the sub-scale of Restrained behavior [64]. Each item represents an eating behavior, and respondents are asked to indicate the frequency at which they exhibit the behavior on a 5-point Likert scale (1 = Never, 5 = Very often). Two outcome measures are obtained by averaging frequency scores of items that assess the same sub-scale. The reliability of the psychometric properties of the DEBQ-R has been demonstrated by several studies, including [65].

#### 2.1.4. Impulsivity Assessment

The fourth section of the survey assessed impulsivity traits through a short version of the Urgency, Premeditation, Perseverance, Sensation Seeking, and Positive Urgency questionnaire (UPPS-P) [66]. The UPPS-P questionnaire is a widely used, self-reported scale to measure individuals’ impulsivity. Since the original UPPS-P scale can be time-consuming, in this study, we employed its short version SUPPS-P [67,68] in the Italian translation developed by [69]. Despite being shorter, the SUPPS-P scale exhibits strong correlations with the full UPPS-P questionnaire and retains similar psychometric properties [68]. The SUPPS-P questionnaire consists of 20 items and, like the UPPS-P, it is divided into five sub-scales that are intended to measure different features of personality traits related to impulsive behavior. The sub-scales are: (1) Negative Urgency, which relates to the tendency to mitigate negative emotions with impulsive behavior (i.e., When I feel bad, I will often do things I later regret in order to make myself feel better now); (2) Positive Urgency, which refers to the tendency to react impulsively to intense positive emotions (i.e., I tend to act without thinking when I am really excited); (3) Absence of Premeditation, which concerns the tendency to behave without thinking on the repercussions of the action taken (i.e., I like to stop and think things over before I do them); (4) Lack of Perseverance, which measures the reduced ability to stay focused on a duty when it is perceived as boring or difficult (i.e., Once I get going on something I hate to stop); (5) Sensation Seeking, which pertains to the tendency to seek risky activities and openness to experience new things (i.e., I would like to learn to fly an airplane). The SUPPS-P questionnaire asks respondents to rate their level of agreement with a number of statements (from strong agreement to strong disagreement) using a 4-point Likert scale. Four items for each sub-scale are included in the questionnaire. After inverting the scores of some questions—so that higher scores always correspond to more impulsive traits—scores are averaged to provide a single outcome measure for each of the five sub-scales.

#### 2.1.5. Income

At the end of the survey, a non-mandatory question asked participants to categorize their total, pre-tax annual income as Low (Up to €28,000), Medium (€28,000–50,000), or High (Above €50,000).

### 2.2. Data Preparation

#### 2.2.1. Calorie Levels

We obtain the total calories in the meal ordered by a participant by summing the calories of the participant’s selected main, side, and dessert dishes. There is a limited number of meals (specifically, 64) that could be chosen by combining the four available choices of main, side, and dessert, and a limited number of values for the total calories in the participant’s selected meal. Thus, as in a real scenario, participants could use the calorie information to distinguish low-calorie dishes from higher-calorie dishes, but they were not shown the exact number of calories in their meal. Considering that the aim of the study was to analyze the overall caloric intake of the meal, rather than the specific serving choice, respondents’ answers have been grouped in four ordered levels according to the total caloric intake estimated from participants’ choice:

- Low (787–950 kcal): all meals including one of the two lowest-calorie mains, the lowest-calorie side and any dessert except the highest-calorie dessert.
- Medium (951–1350 kcal): all meals including exactly two of: one of the two lowest-calorie mains; the lowest-calorie side; any dessert except the highest-calorie dessert.
- High (1351–1675 kcal): all meals including: one of the two highest-calorie mains, one of the two highest-calorie sides and not the highest-calorie dessert; or, one of the two highest-calorie mains, the highest-calorie dessert and not the highest-calorie side; or, one of the two lowest-calorie mains, the highest-calorie dessert and not the lowest-calorie side.
- Very High (1676–1963 kcal): all meals including one of the two highest-calorie mains, one of the two highest-calorie sides and the highest-calorie dessert.

**Figure 2:**
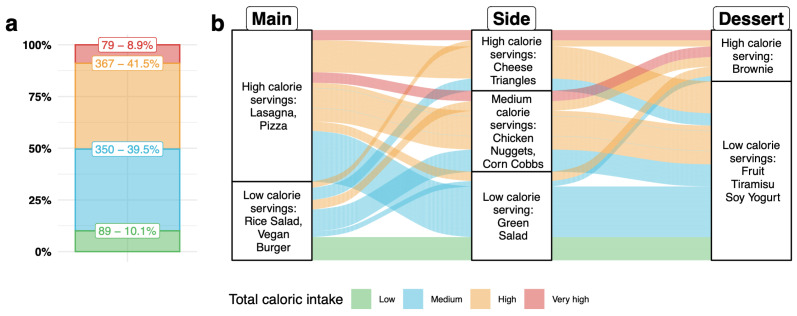
Plot ((**a**); **left**): Histogram showing the prevalence of the four levels of calorie intake corresponding to participants’ selected meals. Plot ((**b**); **right**): Alluvium plot illustrating the distribution of participants’ choices for each of the three courses as well as across courses, coloring meals by their calorie level.

A visual summary of meals corresponding to each calorie level can be seen in Figure 2. Notice that calorie levels are therefore an ordinal response variable, and that a change in the response variable is interpreted as a change in the level of calories in the meal ordered by a participant.

#### 2.2.2. Age, BMI, Education and Nutrition-Related Variables

Participants’ age was merged into six groups for descriptive statistics according to range (18–30, 31–40, 41–50, 51–60, 61–70, 70+) and for all further analysis was considered as a continuous value. From the height and the weight of participants, we derived the BMI, which was then classified according to the standard categories developed by the World Health Organization [70] and labeled as Underweight (BMI < 18.5), Normal (BMI ≥ 18.5 and <25), Overweight (BMI ≥ 25 and <30) or Obesity (BMI ≥ 30). Further, levels of education were merged into two groups: High school or below (answers Lower than high-school diploma or High-school diploma) and Bachelor or above (answers Bachelor’s degree, Master’s degree, or Doctorate). Eating-out frequency levels were merged into three categories: Rarely or never (answers Never or Rarely); Up to 2 times a week (answers 1–3 times per month or 1–2 times a week); and, 3 or more times a week (answers 3–4 times a week or 5 or more times a week). Levels of nutrition knowledge were re-labeled to combine five categories into three: Low (answers Not at all informed and Low informed); Medium (answer Moderately informed); High (answer Completely informed and Very informed). Finally, dietary profiles of respondents were summarized into four larger groups based on the frequency and amount of meat consumption: Does not eat meat (answers Vegetarian or Vegan); Eats little meat (answers Flexitarian and Pescetarian); Eats meat (answer Omnivorous); Other.

### 2.3. Data Analysis

#### 2.3.1. Statistical Methodology

Recall that the first aim of this study is to assess the effect of how calories are displayed (i.e., of the menu design) on the level of calories consumed (i.e., Low, Medium, High or Very-high calories, as defined in Section). This study also aims to assess how the level of calories consumed is potentially affected by an interaction between gender of participant and menu design, or by an interaction between nutrition knowledge of participant and menu design.

Considering that the response is an ordinal categorical variable, we address those aims by fitting the following four ordinal logistic regression models [71], with proportional odds assumption and logit link function [72]:

- Model 1: regresses calorie levels of chosen meals only on the randomly- assigned menu design and on intercepts.
- Model 2: extends Model 1 to include, as controls, demographic characteristics (except nationality), BMI, nutrition-related variables, income, DEBQ-R scores and SUPP-S scores.
- Model 3: extends Model 2 to include the interaction of gender with the randomly assigned menu design.
- Model 4: extends Model 2 to include the interaction of declared nutrition knowledge with the randomly assigned menu design.

For Models 2–4, observations with levels of categorical variables that were not exhibited by at least 20 observations in the dataset were excluded from the analyses. This included responses of participants who identified their gender as neither female nor male (selecting the option Prefer not to say), and individuals who specified their diet as Other (See Table 1).

To ensure the validity of the statistical procedure and the absence of spurious effects, residual plots estimates adopting jitter method were analyzed for each model, checking their normality with qqplots R [73] package and the correct accounting of the factors by the models using residuals versus covariate plots. These materials are available in Supplementary Materials S4. Additionally, all main effects and interaction terms were evaluated for multicollinearity using Generalized Variance Inflation Factors (GVIFs).

To summarize the results of the models, we report the values of the estimated regression coefficients and their associated *p*-values, for each level of the categorical variables included in the analysis (net of the reference level) and for each continuous variable. All statistical analyses used an alpha level of 0.05.

#### 2.3.2. Sensitivity Analyses

To assess the robustness of our results with respect to the choice of grouping total calories into four ordinal levels, the same analyses were repeated using linear models with the number of total calories in participants’ selected meals as response variable. Results are reported in Supplementary Materials S5.

#### 2.3.3. Statistical Software

All analyses and plots were obtained using the statistical software R [73], version 4.4.2. For the main statistical analyses, the function vglm() from the package VGAM [74] was used to fit vector generalized linear models and the package sure [75] was used for residual estimation and analysis. For plotting results, the packages ggplot2, ggpubr [76] and ggalluvial [77] were used. Code and data are available in a dedicated github repository to ensure repeatability of the analyses.

## 3. Results

### 3.1. Participants’ Characteristics

For each of the three randomly assigned menu designs, Table 1 summarizes the characteristics of study participants that were self-reported in the second section of the survey.

Demographics were similar across participants in the three menu-design groups, ranging from 18 to 80, with an average value of 44 years old. Most participants were young adults between 18 and 30 years old, or middle-aged adults between 50 and 60 years old. Adults over 70 years old represented less than five percent of all study participants. Age composition was similar across menu-design groups, with the exception of a slightly higher prevalence of young adults aged 30–40 in the group randomized to menus with PACE labels. Females represented between 57.9 and 58.4 percent of study participants in each menu-design group. Less than one percent of participants in each group chose not to disclose their gender and none of them selected the option Other. Overall, no statistically significant differences were observed among the three menu groups for the variables reported in Table 1.

With respect to education, more than 57 percent of participants in each menu-design group completed at least a Bachelor’s degree. Across menu-design groups, the most commonly chosen location by participants was a city (over 54 percent), followed by the countryside (between 23.1 and 29.9 percent) and, less commonly, the suburbs (less than 17 percent). The most common income level was low (between 43.7 and 51.5 percent), followed by medium (27.5 to 33.1 percent) and high (13.7 to 21.4 percent). Relative to the other menu-design groups, participants in the PACE group were less likely to declare a low income and more likely to declare a high income (possibly a reflection of the slightly different age composition). Between 9 and 12 percent of participants in each menu-design group reported that they are adopting weight-loss diets. Responses about nationality indicate that the vast majority of participants were Italian (98 percent in total and over 96 percent in each menu-design group).

Across all menu-design groups, the majority of participants reported weight and height corresponding to a normal BMI (over 59%), followed by overweight (23.7% to 25.8%). The remaining participants were almost equally distributed between underweight and obesity categories. Meat consumption was also similar across menu-design groups, with more than 70 percent of participants declaring regular meat consumption (i.e., omnivorous), roughly a fifth of participants declaring low meat consumption (i.e., pescetarian or flexitarian diet) and a small (2.3–4.1) percentage of respondents following a diet with no meat (i.e., vegetarian or vegan). Most participants in each menu-design group (over 78 percent) indicated a tendency to eat out up to 2 times a week. Finally, participants who declared a medium nutrition knowledge (i.e., 3 points on a 5-point Likert scale) represented between 45 and 49 percent, followed by 32–38 percent declaring high nutrition knowledge (i.e., 4 or 5 points), and the remaining 15–18 percent declaring low nutrition knowledge (i.e., 1 or 2 points).

### 3.2. Calorie Levels and Composition of Participants’ Selected Meals

The overall prevalence of the four calorie levels among participants’ selected meals is shown in Figure 2a. Most participants selected Medium or High-calorie meals, which together accounted for 81.4% of responses. Low-calorie and Very-high-calorie meals were chosen, respectively, by 9.8% and 8.8% of participants. Relationship between serving choices and the corresponding total caloric level is reported in the mosaic plot in Supplementary Materials S2.

Figure 2b shows which meal compositions were more or less frequently chosen by participants, distinguishing between high-, medium- and low-calorie dishes for each course. Inspecting separately each serving, the most selected choices were High-caloric choices for main dishes (66% of respondents), Medium-caloric choices for sides (38.4% of participants), and low-caloric choices for dessert (78% of participants). Examining the combinations of dishes across the three courses, the most commonly chosen combination was a medium-calorie meal composed of a high-calorie main dish and low-calorie side and dessert. Results reporting individual serving choices combinations are reported in the alluvium plot in Supplementary Materials S3.

### 3.3. Factors Affecting the Chance of Ordering Higher-Calorie Meals

Table 2 reports the results of the four ordinal regression models of calorie levels in meals selected by study participants. A reverse cumulative logit was used to model the change in the log-odds of ordering a meal of a higher calorie level instead of a lower-calorie meal. Intercepts represent estimated: log-odds of ordering a meal with medium or higher calories instead of a low-calorie meal (Medium or higher calories); log-odds of ordering a meal with high or very-high calories instead of a medium or low-calorie meal (High or very high calories); and log-odds of ordering a meal with very-high calories instead of meals with fewer calories (Very high calories). Multicollinearity was low across all predictors: the largest GVIF (adjusted for degrees of freedom) was 1.78 for the interaction between nutritional knowledge and menu design.

When modeling the calorie level of the chosen meal as a function of menu design alone (Model 1), being randomly assigned to the PDI pie chart did not significantly affect the log-odds of ordering higher calorie levels (estimated log-odds increase, 0.134; 95% CI, −0.167 to 0.435) nor did being assigned to the PACE menu (estimated log-odds increase, 0.134; 95% CI, −0.45 to 0.147); these results were consistent across models with additional predictors. Additionally, models with interactions indicated no significant moderation of gender or nutrition knowledge on the effect of PDI pie chart and PACE menus on the calorie levels ordered.

Considering socio-demographic factors, all models including participants’ age estimated a negative and significant coefficient (estimated log-odd decrease: no interaction model: −0.27; 95% CI, −0.37 to −0.016; gender interaction model: −0.27; 95% CI: −0.37 to −0.016; nutrition knowledge interaction model: −0.26; 95% CI, −0.36 to −0.015), suggesting that the chance of choosing higher calorie levels decreases with age. Overweight and obese BMI levels were associated with increased frequency of higher-calorie choices (estimated log-odd increase for overweight: no interaction model: 0.449; 95% CI, 0.12 to 0.77; gender interaction model: 0.448; 95% CI: 0.12 to 0.77; nutrition knowledge interaction model: 0.444; 95% CI, 0.11 to 0.77; estimated log-odd increase for obese: no interaction model: 0.530; 95% CI, 0.006 to 1.05; gender interaction model: 0.531; 95% CI: 0.007 to 1.05; nutrition knowledge interaction model: 0.509; 95% CI, −0.01 to 1.05), while underweight BMI levels were associated with lower log-odds of ordering a higher-calorie meal (estimated log-odd decrease: no interaction model: −0.852; 95% CI, −1.44 to −0.26; gender interaction model: −0.849; 95% CI: −1.44 to −0.26; nutrition knowledge interaction model: −0.858; 95% CI, −1.49 to −0.30). Meat consumption was also a significant predictor across all models that included it, as people declaring not to consume meat at all, or less frequent consumption of meat, appeared more likely to order lower-calorie servings (estimated log-odd decrease for not consuming meat: no interaction model: −1.207; 95% CI, −2.00 to −0.41; gender interaction model: −1.207; 95% CI: −2.00 to −0.41; nutrition knowledge interaction model: −1.283; 95% CI, −2.08 to −0.48; estimated log-odd decrease for less consumption of meat: no interaction model: −0.778; 95% CI, −1.11 to −0.44; gender interaction model: −0.777; 95% CI: −1.11 to −0.44; nutrition knowledge interaction model: −0.762; 95% CI, −1.09 to −0.42).

In our models, eating-out frequency, income level, hunger level, and nutrition knowledge were not significantly associated with the log-odds of ordering higher-calorie meals. Conversely, respondents who declared to follow a weight-loss diet appeared less likely to choose higher-calorie meals, showing consistency between the information reported in the questionnaire and their choices from the menu. Almost all behavioral traits captured by DEBQ and S-UPPS questionnaires showed no significant effects on the caloric content of their chosen meal. The only exception was the restrained eating behavior assessed by the DEBQ: higher scores of restrained eating behavior were associated with a lower probability of higher-calorie choices.

## 4. Discussion

While confirming the effects of known correlates of calorie intake (e.g., BMI, dietary habits), the results of this study call into question the effectiveness of qualitative menu labeling formats compared to standard calorie-only labels. Across all estimated models, neither the PACE labels nor the PDI pie chart had a significant impact on the level of calories ordered. This suggests that alternative labeling strategies may not systematically influence consumers’ behavior—a conclusion that aligns with the results of existing meta-analyses [26]. Furthermore, the interactions between menu designs and respondents’ gender or nutritional knowledge were also non-significant, highlighting that different approaches of presenting calorie content were not sufficient to influence eating behaviors. These findings align with prior research questioning the standalone effectiveness of menu calorie labeling in altering food choices [15].

Given the growing interest in developing more intuitive and engaging nutritional labels, our findings raise critical questions about why alternative formats fail to produce measurable behavioral changes. One possibility is that consumers may not fully understand or engage with these labels, as suggested by previous research on cognitive processing of nutrition information [40]. Future studies should explore whether additional contextual cues, such as interactive menu features or real-time feedback, could enhance label comprehension and impact. Furthermore, it will be important to consider the results obtained in countries that have adopted clear policies on nutritional labeling and implemented them robustly [78,79].

PACE labels have been told to encourage consumers to purchase lower-calorie items on menus [32] and lead to a reduction in high-calorie food purchases [30,31]. However, our study did not find any of these effects, in line with findings from [35] and [34]. As suggested by [80], PACE label may not constitute an effective labeling strategy to reduce food consumption.

Similarly, the PDI pie chart did not demonstrate superior performance compared to the standard numeric calorie count, consistent with the findings of [39]. One possible explanation, as suggested by [40], is that respondents may not have understood how to use the pie charts to judge the amount of calories relative to recommended intakes.

Contrary to what is reported in the literature, we did not find any effect of gender on calorie selection based on menu labeling [22,24,28,81]. Women did not order fewer calories than men, and the lack of significant interactions between menu designs and respondent gender suggests that different menu designs did not have a differential impact on dietary choices across specific subgroups.

The way in which calorie information is conveyed does not appear to significantly influence consumer choices, regardless of individuals’ self-assessed nutritional knowledge. Contrary to previous findings [17,28], the caloric information provided in our study did not significantly influence food choices, neither among individuals with limited nutritional knowledge nor those with a more extensive understanding. This suggests, as reported by [82], that the lack of use of nutritional information is not merely a cognitive issue but also involves motivational factors such as personal goals, perceived barriers, and the value placed on making healthier choices. However, these results should be interpreted with caution, as nutrition knowledge in our study was assessed using a single self-reported item, which may not accurately reflect actual knowledge levels. Future research should focus on interventions that translate nutritional awareness into actionable behavior, such as personalized nudges, experiential education, or social influences.

Older participants were less likely to choose high-calorie options, which may reflect a combination of increased health awareness with age [28], as well as physiological changes such as reduced appetite [83] and a natural decline in caloric requirements associated with aging [84]. These findings are consistent with existing literature showing that older adults generally consume fewer calories and may be more health-conscious in their dietary choices. Conversely, individuals with higher BMI were more likely to select calorie-dense menu items, consistent with findings that people with obesity tend to prefer energy-dense foods. Our results partially align with prior research [20] but contrast with [85], who found that nutritional information was more effective for non-overweight individuals. These discrepancies underscore the complexity of dietary decision-making and suggest that personalized interventions may be necessary to effectively target different BMI groups.

The frequency of eating out did not emerge as a significant predictor of caloric choices in our study. A possible explanation lies in the relatively low frequency of eating out among participants, with most reporting dining out no more than twice per week. This occasional dining behavior may lead individuals to pay less attention to calorie intake when eating out, as they might perceive these meals as having a negligible impact on their overall weekly energy balance. In contrast, individuals who eat out more frequently might be more conscious of their daily caloric intake, given the potential cumulative effect on their health.

As suggested by [16], future studies should explore whether eating out frequency moderates the impact of menu labeling on caloric selection, particularly across cultures where dining out is more common. Since dietary habits are shaped by cultural norms and lifestyles, a cross-cultural analysis could reveal how labeling interacts with eating environments. This would enhance our understanding of calorie labeling effectiveness and inform tailored public health strategies.

Participants who reported limiting their meat consumption or identifying as non-meat eaters consistently selected lower-calorie options compared to habitual meat consumers. Although evidence on the influence of dietary patterns on responsiveness to calorie labeling is still limited, prior studies have indicated that non-meat eaters tend to prioritize ethical and health-related motivations in their food choices [86], which may, in turn, increase their attention to caloric information [87]. This alignment between self-reported dietary habits and menu choices highlights the significant role of individual predispositions in shaping food decisions. Furthermore, restrained eating behavior, as measured by the DEBQ, was strongly associated with a higher likelihood of choosing lower-calorie options, consistent with previous findings [81].

However, despite evidence linking impulsivity to overeating, eating disorders, and a preference for high-calorie foods [49,50,88,89], our study did not find a significant effect of impulsivity traits on the caloric content of the servings chosen. This suggests that the influence of impulsivity on food selection may be context-dependent or modulated by other factors, such as the decision-making environment or the availability of external cues. Similar results were observed by [90], who found no significant relationship between impulsivity traits and the consumption of animal-based foods, suggesting that the impact of impulsivity on dietary choices may not be as straightforward as previously assumed.

These findings highlight the critical role of individual traits, such as impulsivity (measured by the SUPPS-P) and pre-existing eating behaviors (measured by DEBQ) in shaping dietary decisions [91]. Self-disciplined behaviors and voluntary dietary restrictions appear to exert a stronger influence on caloric choices than exposure to calorie labeling alone. Furthermore, it is important to acknowledge that broader social and cultural factors (e.g., norms around dining, attitudes toward health and food, social desirability) may also modulate how individuals process and respond to labeling interventions. This underscores the need for public health strategies that integrate both individual-level and contextual approaches—focusing on personal motivations and self-regulation, while also considering environmental and cultural determinants—to achieve more meaningful and sustained impacts on dietary behavior.

Taken together, our findings suggest that, compared to individual characteristics like dietary patterns, restrained eating or BMI, label design may have a negligible impact on calories ordered. It is possible, however, that more proximal contextual variables—such as momentary motivation to eat healthily, whether the meal is perceived as a routine versus special-occasion choice, or the social dining context—may condition how consumers use calorie information. For example, calorie labels may carry greater weight when individuals are actively trying to control their weight, choosing a weekday meal, or eating alone, and be largely disregarded in indulgent or celebratory meals. While our study investigated potential moderator effects of gender and nutritional knowledge, we did not test for a moderating role of situational factors, which were not included in our survey. Future research should incorporate assessments of dining context and motivational state and examine their interaction with labeling to clarify whether qualitative labels can shift caloric selections in specific circumstances.

Despite its contributions, this study also has limitations that should be acknowledged.

First, an inherent limitation of online studies is that participants’ choices were hypothetical and did not involve real purchasing or food consumption. The absence of real economic trade-offs and direct sensory engagement with food (e.g., seeing or smelling it) reduces behavioral realism and may weaken the external validity of our findings. Although hypothetical food selections are commonly used to model food choice, prior research suggests that they may not always align with real-world purchasing decisions, especially when self-control, social influences, or immediate sensory gratification come into play [92]. Thus, our findings should be interpreted with caution. Future research should aim to replicate these findings in experimental restaurant settings where participants are required to pay for their meals.

Second, one methodological limitation is that the menu used in the study contained a relatively small number of options. This constrained choice set may have influenced participants’ selections, as they were limited in their ability to substitute higher-calorie dishes with lower-calorie alternatives. In real-world settings, menus often provide a wider variety of low-calorie options, which could amplify or attenuate the impact of calorie labeling on consumer behavior. Future research should consider testing labeling effects in menu contexts with greater dietary diversity.

Third, participants in this study were required to choose one dish for each course. In reality, however, restaurant customers have more flexibility in ordering, including the option to skip certain courses altogether. This means that a critical mechanism through which calorie labeling may influence behavior— encouraging individuals to forgo high-calorie items, such as desserts—was not captured in this study. Future experiments should allow for a more flexible meal selection process to better reflect naturalistic dining behavior.

Fourth, this study presented calorie information at the level of individual dishes rather than as a cumulative total for meal combinations. Some research suggests that aggregating caloric information may make it more salient and actionable, potentially nudging consumers toward lower-calorie choices [93,94]. However, a recent study found no significant effect of providing total calorie information [95]. Future studies could explore how different formats of calorie aggregation affect consumer decision-making.

Fifth, we acknowledge that cultural factors may influence how consumers process and respond to menu labeling. It is therefore plausible that an Italian sample—due to its specific food culture, dietary patterns, and attitudes toward health—might have responded differently from samples in other countries to the same menu labeling formats. This aspect represents a potentially important moderator and should be addressed explicitly in future cross-cultural studies.

Moreover, we did not collect qualitative data on participants’ perceptions of the labels (e.g., perceived helpfulness, clarity, or salience) or on their decision-making strategies while browsing the menu. Such information would have allowed a richer interpretation of the null effects by clarifying whether the labels were unnoticed, poorly understood, or simply considered unimportant in the decision process. Future studies should adopt mixed-methods designs that combine quantitative behavioral outcomes with open-ended questions, think-aloud procedures, or post-choice interviews to better capture the mechanisms underlying responses to menu labeling.

Finally, the sample in this study had a relatively high education level, which may limit the generalizability of the findings.

## 5. Conclusions

This study demonstrates that while sociodemographic factors (e.g., age, BMI) and individual traits (e.g., diet, restrained eating) significantly influence caloric intake, alternative menu labeling formats such as PACE labels and PDI pie charts do not appear to be inherently more effective than standard calorie-only labels. The presumed advantages of interpretive or symbolic labels in enhancing comprehension and promoting healthier choices may not apply universally, at least within the context of this study. One plausible explanation is that the additional information conveyed by PACE and PDI labels may have had relatively low salience and could have been overlooked. Moreover, the extra symbols and graphics may have introduced a modest increase in cognitive load, which, combined with limited motivation to eat healthily in a “treat-like” fast-food context, may have led participants to rely primarily on taste and habit rather than on calorie information when making their selections. These findings challenge the assumption that qualitative labels are inherently superior to numeric calorie counts and underscore the need for further research to clarify the mechanisms driving their influence on consumer behavior.

To foster meaningful dietary changes, interventions must extend beyond calorie labeling by incorporating educational and behavioral strategies tailored to individual characteristics. While providing calorie information can raise awareness, it does not necessarily translate into healthier choices. Future efforts should consider integrating labeling strategies with complementary interventions, such as public health campaigns, interactive tools, and behavioral nudges, to enhance their effectiveness. Additionally, understanding how contextual and cultural factors interact with menu labeling will be crucial for designing more impactful public health policies.

Another important aspect that warrants greater attention is the role of taste intuition in food decision-making. As highlighted by [96], taste intuition (e.g., the belief that higher-calorie foods are more palatable) can strongly influence consumer preferences and may override nutritional considerations, even when calorie or sodium information is provided. Therefore, future studies should assess how hedonic perceptions mediate or moderate the impact of menu labeling and explore strategies to align health-related messages with consumers’ taste expectations.

In conclusion, while menu labeling remains a valuable tool for promoting dietary awareness, its impact on consumer behavior appears limited when used in isolation. Further studies should test labeling effectiveness in real dining environments and examine cross-cultural differences in how calorie information is perceived and utilized, as dietary norms and food literacy levels vary across populations. Thus, future interventions should adopt a multifaceted approach that accounts for individual differences, decision-making contexts, and behavioral strategies to more effectively support healthier eating habits.

## Figures and Tables

**Table 1:** Characteristics of study participants. Columns 1–3 group participants according to their randomly assigned menu design and show percentages (in brackets) for the total number of participants in each group. Column 4 combines participants across all groups and shows the total study participants’ percentages (in brackets).

Characteristics	Only Calories	PACE	PDI Pie Chart	Total
*Age*				
18–30	86 (28.8%)	65 (22.0%)	78 (26.8%)	229 (25.9%)
31–40	38 (12.7%)	55 (18.7%)	36 (12.4%)	129 (14.6%)
41–50	62 (20.7%)	58 (19.7%)	55 (18.9%)	175 (19.8%)
51–60	71 (23.8%)	75 (25.4%)	71 (24.4%)	217 (24.5%)
61–70	35 (11.7%)	34 (11.5%)	38 (13.0%)	107 (12.1%)
70+	7 (2.3%)	8 (2.7%)	13 (4.5%)	28 (3.1%)
*Gender*				
Female	173 (57.9%)	171 (58.0%)	170 (58.4%)	514 (58.1%)
Male	125 (41.8%)	123 (41.7%)	119 (40.9%)	367 (41.5%)
Prefer not to say	1 (0.3%)	1 (0.3%)	2 (0.7%)	4 (0.4%)
*Education*				
High school or below	118 (39.5%)	108 (36.6%)	123 (42.3%)	349 (39.4%)
Bachelor or above	181 (60.5%)	187 (63.4%)	168 (57.7%)	536 (60.6%)
*Location*				
Countryside	69 (23.1%)	83 (28.1%)	87 (29.9%)	239 (27.0%)
Suburbs	47 (15.7%)	50 (17.0%)	46 (15.8%)	143 (16.2%)
City	183 (61.2%)	162 (54.9%)	158 (54.3%)	503 (56.8%)
*Income level*				
Low	144 (48.2%)	129 (43.7%)	150 (51.5%)	423 (47.8%)
Medium	99 (33.1%)	93 (31.5%)	80 (27.5%)	272 (30.7%)
High	41 (13.7%)	63 (21.4%)	47 (16.2%)	151 (17.1%)
Not declared	15 (5.0%)	10 (3.4%)	14 (4.8%)	39 (4.4%)
*Nationality*				
Italy	288 (96.3%)	292 (99.0%)	287 (98.6%)	867 (98.0%)
Other	11 (3.7%)	3 (1.0%)	4 (1.4%)	18 (2.0%)
*BMI level*				
Underweight	21 (7.0%)	17 (5.7%)	13 (4.5%)	51 (5.8%)
Normal	178 (59.5%)	182 (61.7%)	192 (66.0%)	552 (62.4%)
Overweight	77 (25.8%)	71 (24.1%)	69 (23.7%)	217 (24.5%)
Obesity	23 (7.7%)	25 (8.5%)	17 (5.8%)	65 (7.3%)
*Diet*				
Does not eat meat	7 (2.4%)	12 (4.1%)	5 (1.7%)	24 (2.7%)
Eats little meat	56 (18.7%)	65 (22.0%)	57 (19.6%)	178 (20.1%)
Eats meat	232 (77.6%)	217 (73.6%)	226 (77.7%)	675 (76.3%)
Other	4 (1.3%)	1 (0.3%)	3 (1.0%)	8 (0.9%)
*Weight-loss diet*				
Yes	36 (12.0%)	36 (12.2%)	27 (9.3%)	99 (11.2%)
No	263 (88.0%)	259 (87.8%)	264 (90.7%)	786 (88.8%)
*Eating-out frequency*				
Rarely or Never	52 (17.4%)	50 (16.9%)	47 (16.2%)	149 (16.8%)
Up to 2 times a week	234 (78.3%)	228 (77.3%)	232 (79.7%)	694 (78.4%)
3 or more times a week	13 (4.3%)	17 (5.8%)	12 (4.1%)	42 (4.8%)
*Nutritional knowledge*				
Low	45 (15.0%)	47 (15.9%)	53 (18.2%)	145 (16.4%)
Medium	148 (49.5%)	134 (45.4%)	144 (49.5%)	426 (48.1%)
High	106 (35.5%)	114 (38.7%)	94 (32.3%)	314 (35.5%)

**Table 2:** Results of ordinal, reverse-cumulative regression models with logit link function. Significant estimates (*p*-value < 0.05) are bolded. ‘*’ indicates a *p*-value between 0.05 and 0.01, ‘**’ indicates a *p*-value between 0.001 and 0.01, ‘***’ indicates a *p*-value lower than 0.001.

Factors	Only Menu Model (Pseudo R^2^: 0.76)		All Factors Without Interactions (Pseudo R^2^: 0.81)		With Gender: Menu Interaction (Pseudo R^2^: 0.81)		With Nutrition Knowledge: Menu Interaction (Pseudo R^2^: 0.81)	
	Estimate	Std. Error	Estimate	Std. Error	Estimate	Std. Error	Estimate	Std. Error
*Intercepts*								
Medium or higher calories	2.203 ***	**0.142**	4.153 ***	0.71	4.152 ***	**0.712**	4.292 ***	**0.726**
High or very high calories	0.022	0.11	1.718 *	0.696	1.716 *	**0.698**	1.849 **	**0.712**
Very high calories	−2.321 ***	**0.147**	−0.87	0.697	−0.872	0.7	−0.753	0.713
*Menu Designs*								
PDI pie chart	0.134	0.154	0.168	0.16	0.176	0.21	0.178	0.275
PACE	−0.155	0.153	−0.098	0.159	−0.12	0.208	−0.379	0.262
*Socio-Demographics*								
Gender (Male)			−0.033	0.157	−0.044	0.24	−0.039	0.157
Age			−0.027 ***	**0.005**	−0.027 ***	**0.005**	−0.026 ***	**0.005**
*Instruction Level*								
High school or below			−0.005	0.143	−0.004	0.144	−0.015	0.144
*Income Levels*								
Low			0.055	0.211	0.057	0.212	0.03	0.212
Medium			−0.125	0.203	−0.123	0.203	−0.13	0.204
Not declared			0.177	0.367	0.18	0.367	0.217	0.368
*BMI Level*								
Underweight			−0.852 **	**0.302**	−0.849 **	**0.302**	−0.898 **	**0.304**
Overweight			0.449 **	**0.167**	0.448 **	**0.167**	0.444 **	**0.167**
Obesity			0.530 *	**0.267**	0.531 *	**0.267**	0.509.	0.268
*Meat Consumption*								
Eats little meat			−0.778 ***	**0.17**	−0.779 ***	**0.17**	−0.762 ***	**0.17**
Does not eat meat			−1.207 **	**0.406**	−1.207 **	**0.406**	−1.283 **	**0.408**
*Eating out frequency*								
Up to 2 times a week			−0.143	0.314	−0.141	0.314	−0.148	0.315
Rarely or never			−0.018	0.354	−0.017	0.354	−0.006	0.355
*Hunger Level*								
Medium			0.267	0.222	0.268	0.223	0.265	0.223
High			0.116	0.183	0.116	0.183	0.127	0.184
*Nutrition Knowledge*								
Medium			0.171	0.147	0.172	0.147	0.117	0.249
Low			0.279	0.209	0.28	0.209	−0.142	0.349
*Weight-loss diet*								
Yes			0.508 *	**0.225**	0.510 *	**0.225**	0.520 *	**0.225**
*DEBQ-R*								
Restricted Behavior			−0.068 ***	**0.019**	−0.068 ***	**0.019**	−0.069 ***	**0.019**
Restricted Intention			0.031	0.038	0.031	0.038	0.034	0.038
*SUPP-S*								
Negative urgency			0.005	0.033	0.005	0.033	0.003	0.033
Positive urgency			0.03	0.039	0.03	0.039	0.035	0.039
Sensation seeking			0.016	0.029	0.016	0.029	0.014	0.029
Perseverance			0.046	0.034	0.046	0.034	0.044	0.035
Premeditation			−0.024	0.041	−0.025	0.041	−0.027	0.041
*Interactions Menu: Gender*								
PDI pie chart: male					−0.02	0.324		
PACE: male					0.053	0.321		
*Interactions Menu: Nutrition Knowledge*								
PDI pie chart: medium							−0.17	0.357
PACE: medium							0.328	0.348
PDI pie chart: low							0.472	0.484
PACE: low							0.826.	0.482

## Data Availability

The original data on all participants who provided their consent, the pre-processing scripts to create the dataset used in our analyses, and the associated codebook for the variables are publicly available in a GitHub repository at https://github.com/federicazoe/calorie-labels-paper.

## Supplementary Materials

The following supporting information can be downloaded at: https://www.mdpi.com/article/10.3390/nu17233642/s1, S1: English translation of the original Italian survey; S2. Alluvium plot resolved for specific serving choice; S3. Mosaic plot linking serving choices and caloric intake level; S4. Residuals of the cumulative ordinal models; S5. Comparison with linear models.

## Abbreviations

The following abbreviations are used in this manuscript:

PACE	Physical Activity Calorie Equivalent
PDI	Percent Daily Intake
BMI	Body Mass Index
DEBQ	Dutch Eating Behavior Questionnaire
SUPPS-P	Short—Urgency, Premeditation, Perseverance, Sensation Seeking and Positive Urgency questionnaire
