# Supplementary material for: The Way Calories Are Displayed on Restaurant Menus May Not Affect Calorie Intake: Evidence from an Online Experiment
Source: Nutrients. 2025 Nov 21;17(23):3642. doi: 10.3390/nu17233642 (PMC12693276; doi:10.3390/nu17233642)
Supplement: Supplementary file 1 [file nutrients-17-03642-s001.zip › nutrients-3976685-supplementary-revised.pdf]

Supplementary materials

S1. English translation of the original Italian survey

1. Informed Consent
2. How hungry are you on a scale from 1 (not at all) to 7 (extremely) right now?
    - Not at all
    - Little hungry
    - Slightly hungry
    - Somewhat hungry
    - Moderately hungry
    - Very hungry
    - Extremely hungry
3. Please consider the menu above. Imagine you are in a restaurant; select the main dish you would order more willingly.
    - Pizza margherita (1)
    - Lasagna (2)
    - Red rice salad (3)
    - Vegan hamburger (4)
4. And which of these side dishes?
    - Mini corn cobs (1)
    - Chicken nuggets (2)
    - Cheesy triangles (3)
    - Green salad (4)
5. Which of these desserts would you order to finish off the meal?
    - Tiramisu (1)
    - Brownie (2)
    - Fruit (3)
    - Soy yogurt (4)
6. How old are you?
7. What gender do you identify with?
    - Male (1)

- Female (2)
- Others (3)
- Prefer not to say (4)

8. How tall are you (centimeters)?
9. How much do you weight (kilograms)?
10. What is the highest level of education you have achieved?

    - Lower than high school diploma (1) … PhD (5)

11. What is your nationality?
12. What country do you live in?
13. Where do you live?

    - City (1)
    - Suburbs (2)
    - Countryside (3)

14. How well informed do you consider yourself in nutritional field?

    - Not informed at all (1)
    - Slightly informed (2)
    - Moderately informed (3)
    - Very informed (4)
    - Completely informed (5)

15. How often do you eat out in a restaurant?

    - Never (1)
    - Rarely (2)
    - Sometimes (1-3/month) (3)
    - 1 to 2 times/week (4)
    - 3 to 4 times/week (5)
    - 5 or more times/week (6)

16. Which of the following options best describe your diet?

    - Vegan (do not eat dairy products, eggs or any other animal product) (1)
    - Vegetarian (do no eat any meat, poultry, game, fish or shellfish) (2)
    - Flexitarian/plant-based (mainly vegetarian but occasionally eat meat or fish) (3)
    - Pescetarian (eat fish but do not eat meat or poultry) (4)
    - Omnivore (eat both meat and fish) (6)
    - Other (7)

17. Are you following a diet for weight loss at the moment?

    - No (1)
    - Yes (2)

18. DEBQ: Please, indicate on a scale from 1 (never) to 5 (very often) how much you agree with the following statements

    1. If you gain weight, do you try to eat less than usual?
    2. During meals, do you try to eat less than you'd like?
    3. How often do you refuse foods or beverages offered to you because you worry about your weight?
    4. Do you give a lot of attention to what you eat?
    5. Do you choose diet foods volountarily?
    6. If you happen to eat too much, do you eat less than usual on the following day?
    7. Do you eat less volountarily in order not to gain weight?
    8. How often do you try not to eat in between meals because you are watching your weight?
    9. How often do you try to avoid eating in the evening because you are watching your weight?
    10. How much do you connect your weight with what you eat?

19. (SUPPS-P) Please, indicate on a scale from 1 (strongly disagree) to 4 (strongly agree) how much you agree with the following statements

    1. I usually reflect attentively before acting.
    2. When I am really excited, I tend not to think about the consequences of my actions. 3.Sometimes I like doing scary things.
    3. When I am nervous, I often act without reflecting.
    4. I generally like following through with things until the end.
    5. My way of thinking is usually very attentive and careful.
    6. When discussions get heated, I often say things I regret afterwords.
    7. I finish what I start.
    8. I quite enjoy taking risks.
    9. When I am particularly happy, I feel like I can't stop myself from losing control.
    10. Once I start a project, I almost always finish it.
    11. I often make things worse because when I'm nervous I act without thinking.
    12. I usually make decisions after an accurate reasoning.
    13. I generally look for new and exciting experiences and emotions.
    14. When I am particularly excited, I often act without reflecting.
    15. I am a productive person that always finishes their tasks.
    16. When I feel rejected, I often say things I regret afterwards.
    17. I like to try out new experiences and sensations, even when they are a bit scary and maverick.

18. Before making a decision, I consider all the pros and cons.
19. When I am very happy, it's like for me it's normal to give in to my cravings and indulge in excesses.

20. What was your total gross income in the past year?

- up to € 28,000
- € 28,000 to € 50,000
- over € 50,000

S2. Alluvium plot resolved for specific serving choice

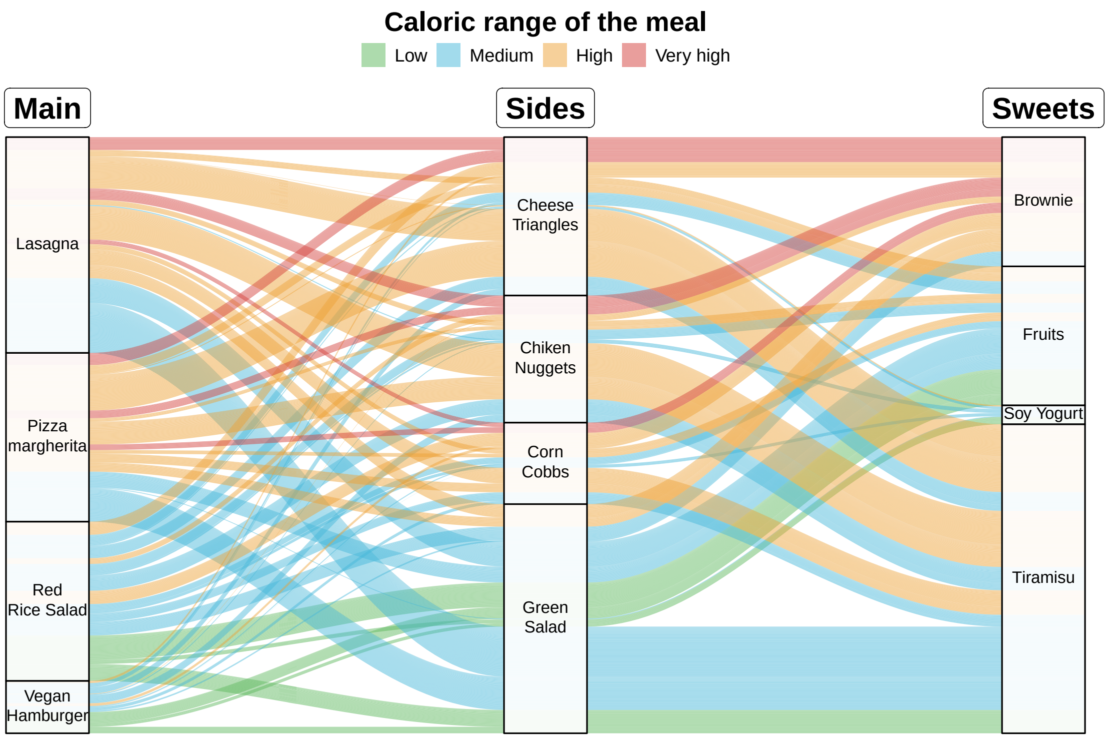

S3. Mosaic plot linking serving choices and caloric intake level

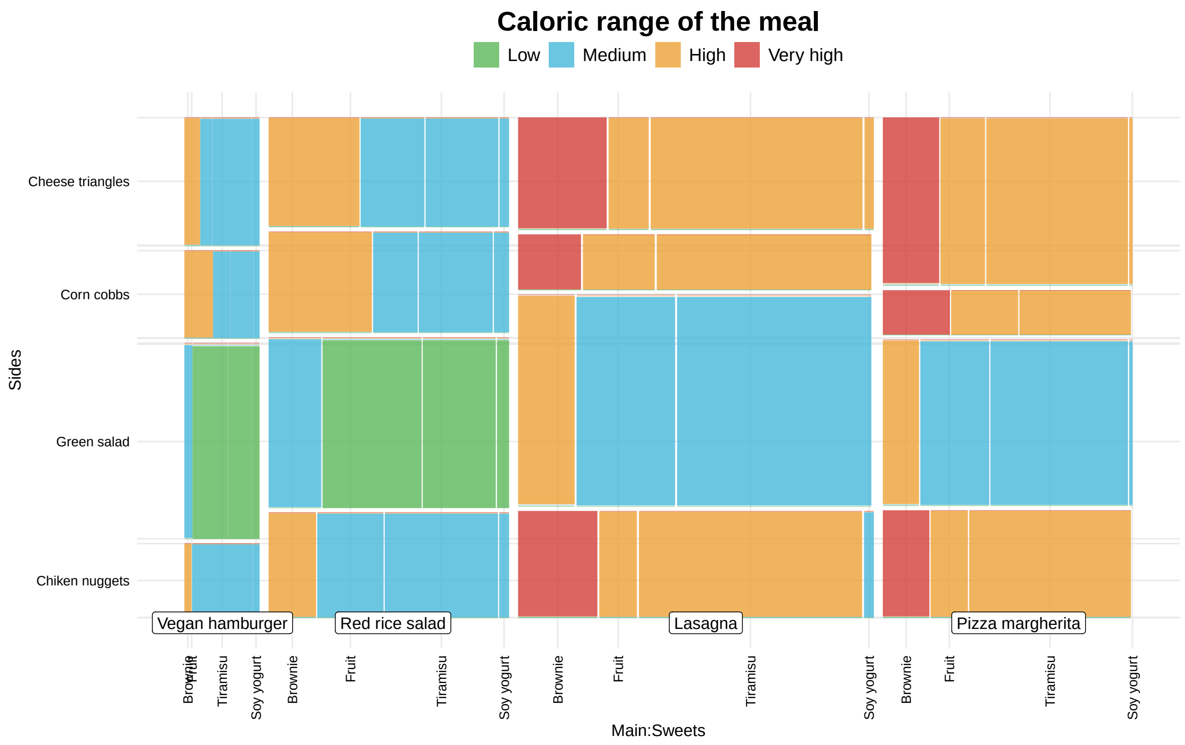

S4. Residuals of the cumulative ordinal models

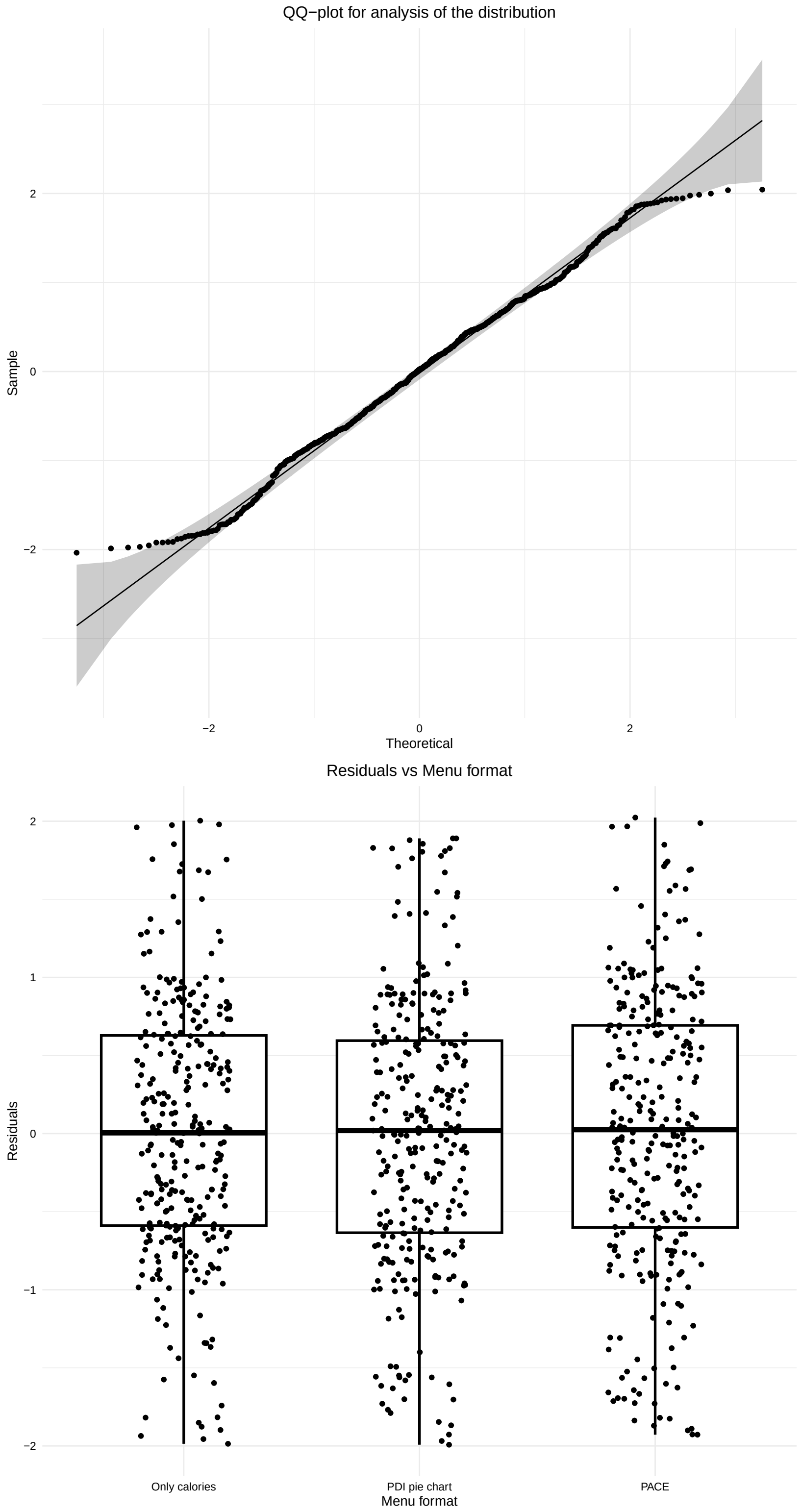

Figure 1: Residuals analysis for Cumulative ordinal model 'Only Menu'

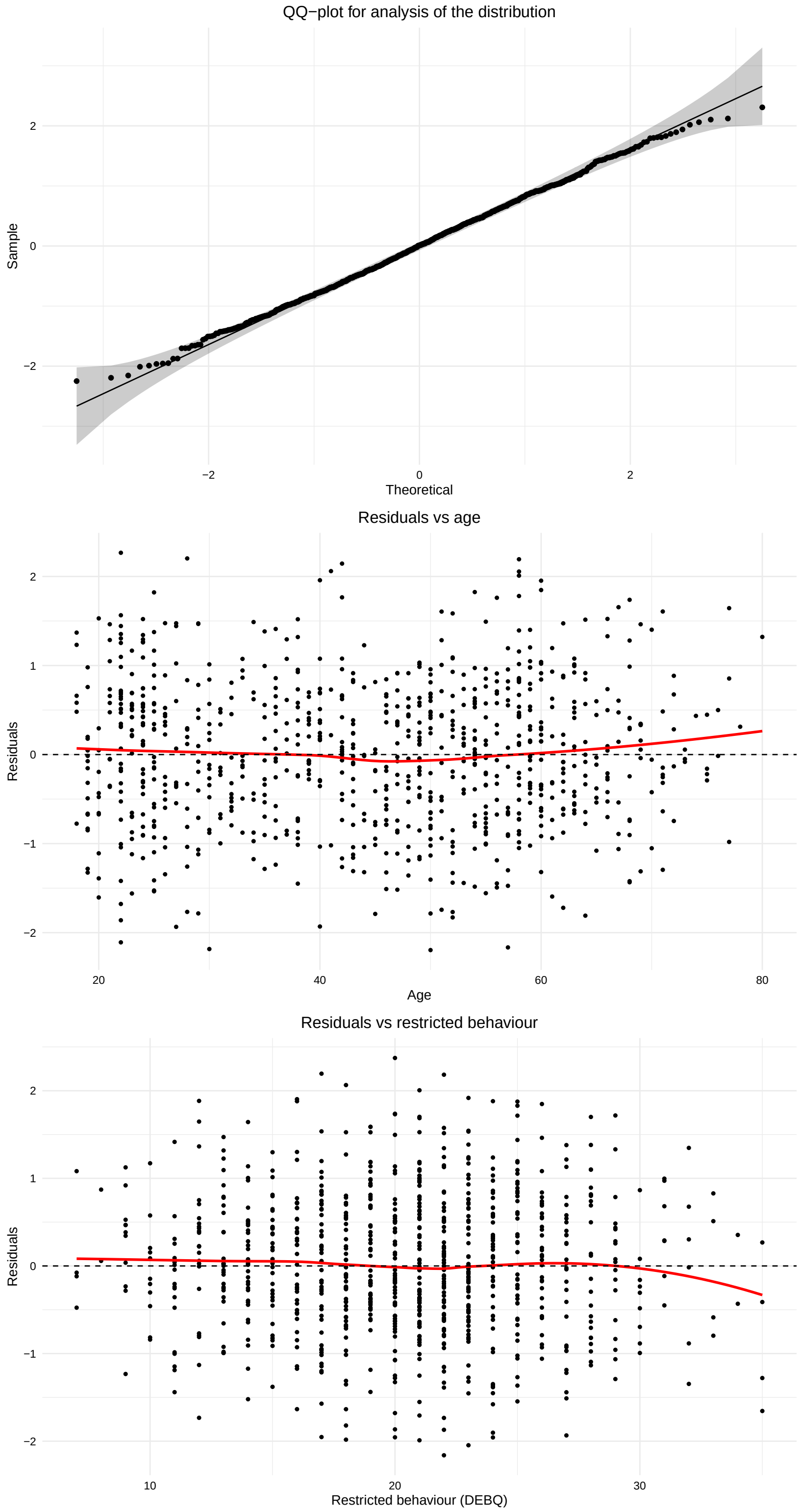

Figure S2: Residuals analysis for Cumulative ordinal model ‘All factors without interaction’

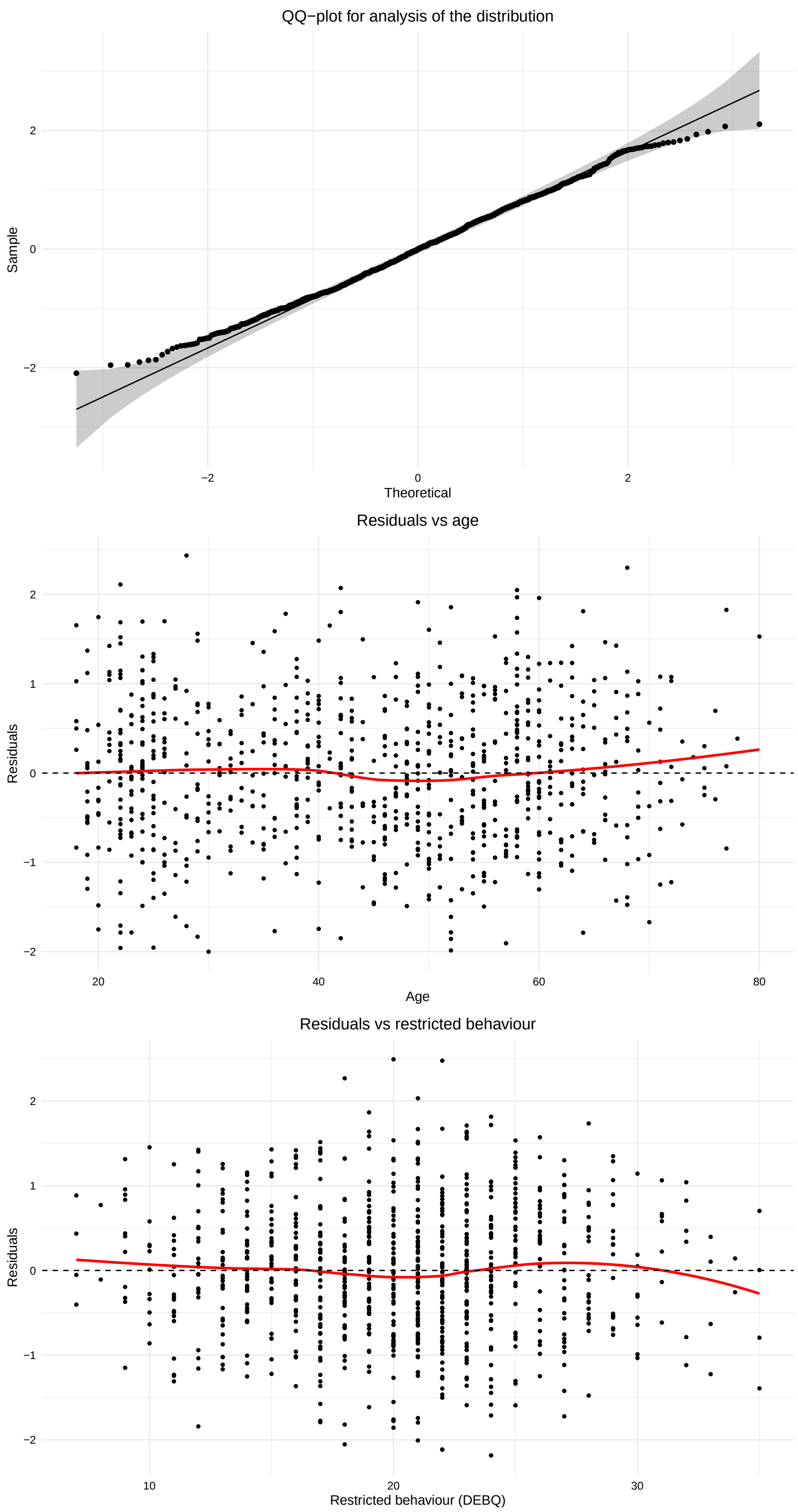

Figure S3: Residuals analysis for Cumulative ordinal model 'With gender:menu interaction'

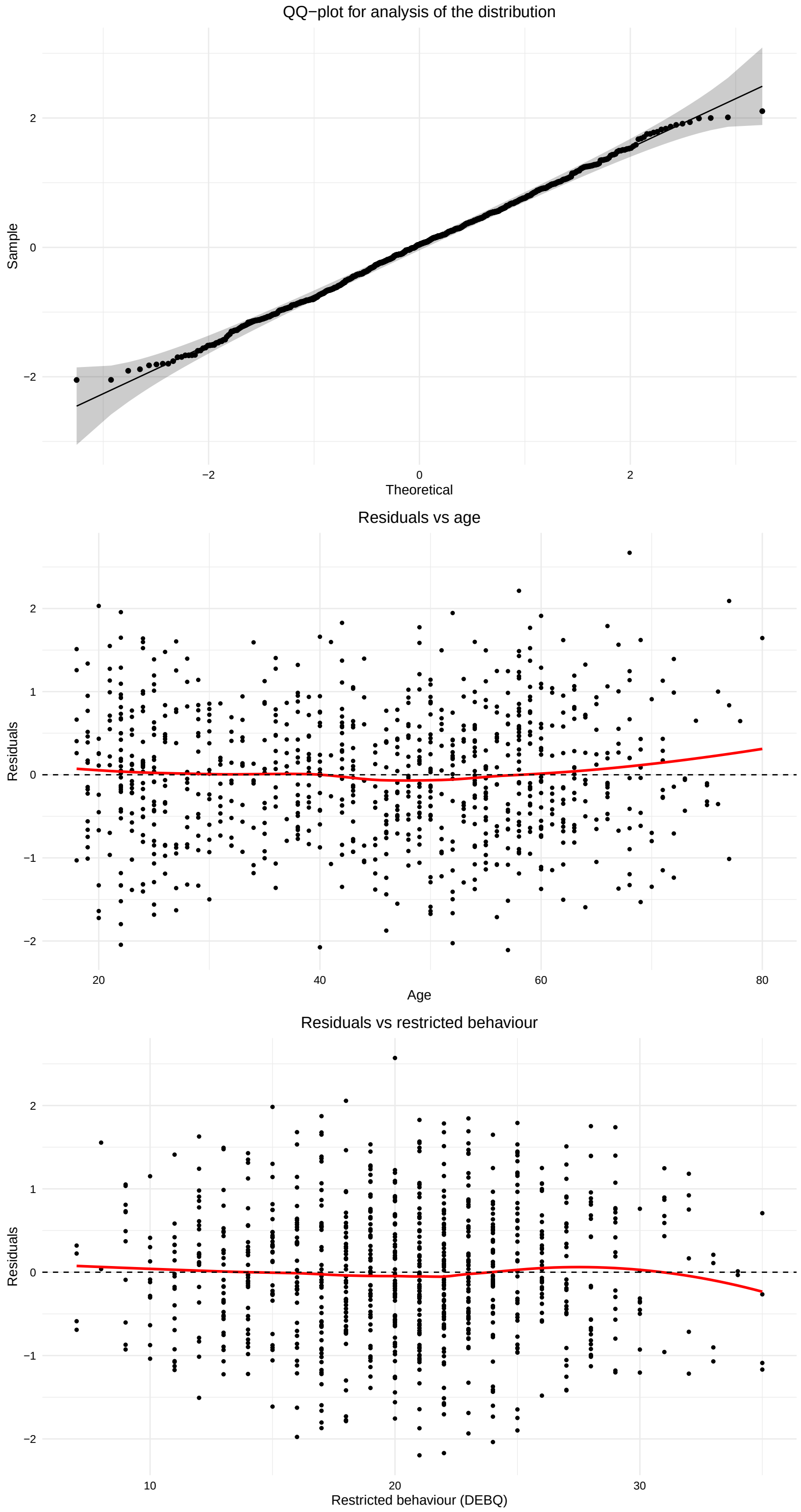

Figure S4: Residuals analysis for Cumulative ordinal model ‘With Nutrition Knowledge:Menu interaction’

S5. Comparison with linear models

Table S1: Overall Results of the linear regression models

	Only Menu model		All factors without interactions		With gender:menu interaction		With Nutrition knowledge:menu interaction	
Factors	Estimate	Std. Error	Estimate	Std. Error	Estimate	Std. Error	Estimate	Std. Error
**Intercepts**								
**(Intercept)**	**1358.441******	**15.632**	**1556.361******	**93.572**	**1554.69******	**94.026**	**1566.184******	**95.634**
**Menu Displays**								
Pie chart	11.689	22.258	13.111	21.518	17.994	28.333	19.077	37.088
Pace	-24.58	22.181	-15.955	21.402	-18.694	28.155	-44.13	35.291
**Socio-Demographics**								
Male			-4.311	21.152	-2.691	32.442	-4.12	21.179
**Age**			**-2.844******	**0.711**	**-2.857******	**0.713**	**-2.779******	**0.713**
**Instruction Level**								
High school or below			-4.152	19.325	-3.833	19.379	-5.399	19.355
**Income Levels**								
Low			21.863	28.486	21.993	28.648	19.533	28.606
Medium			8.015	27.411	8.456	27.508	7.926	27.535
Not declared			46.009	49.397	46.57	49.531	49.743	49.537
**BMI Level**								
**Underweight**			**-108.892*****	**40.521**	**-108.184*****	**40.61**	**-114.554*****	**40.744**
**Overweight**			**56.755***	**22.447**	**56.727***	**22.472**	**55.936***	**22.476**
Obese			65.666.	35.933	66.081.	35.993	64.724.	36.098
**Meat Consumption**								
**Eats little meat**			**-80.842******	**22.675**	**-81.112******	**22.709**	**-78.809******	**22.745**
**Does not eat meat**			**-107.635***	**54.415**	**-107.761***	**54.476**	**-112.616***	**54.634**
**Eating out frequency**								
Up to 2 times a week			-13.392	42.379	-12.848	42.444	-14.646	42.441
Rarely or never			6.369	47.76	7.101	47.858	6.374	47.832
**Hunger Level**								
Medium			30.535	29.863	30.555	30.016	30.657	30.012
High			8.305	24.683	8.218	24.711	10.256	24.722
**Nutrition Knowledge**								
Medium			26.378	19.883	26.538	19.91	24.962	33.631
Low			47.014.	28.137	47.219.	28.184	-0.396	47.158
**Dietary restriction**								
**Yes**			**63.9***	**30.178**	**64.273***	**30.224**	**65.004***	**30.243**
**DEBQ-R**								
**Restricted Behavior**			**-9.686******	**2.529**	**-9.671******	**2.533**	**-9.79******	**2.537**
Restricted Intention			5.604	5.117	5.594	5.123	5.948	5.134
**SUPP-S**								
Negative urgency			1.275	4.493	1.297	4.5	0.966	4.511
Positive urgency			2.583	5.201	2.599	5.207	3.21	5.227
Sensation seeking			3.217	3.871	3.257	3.877	2.898	3.884
Perseverance			3.892	4.629	3.922	4.636	3.542	4.644
Premeditation			-5.062	5.476	-5.158	5.486	-5.108	5.487
**Interactions Menu:Gender**								
Pie chart:male					-11.682	43.771		
Pace:male					6.682	43.446		
**Interactions Menu:Nutrition Knowledge**								
Pie chart:medium							-27.251	48.235
Pace:medium							30.014	47.035
Pie chart:low							47.778	64.973
Pace:low							92.673	64.891
